# COVID-19 and the academy: opinions and experiences of university-based scientists in the U.S.

**DOI:** 10.1057/s41599-021-00823-9

**Published:** 2021-06-17

**Authors:** Timothy P. Johnson, Mary K. Feeney, Heyjie Jung, Ashlee Frandell, Mattia Caldarulo, Lesley Michalegko, Shaika Islam, Eric W. Welch

**Affiliations:** 1 Department of Public Administration, University of Illinois at Chicago, Chicago, IL USA; 2 Center for Science, Technology and Environmental Policy Studies, School of Public Affairs, Arizona State University, Phoenix, AZ USA

**Keywords:** Sociology, Science, technology and society

## Abstract

Much of the available evidence regarding COVID-19 effects on the scientific community in the U.S. is anecdotal and non-representative. We report findings from a based survey of university-based biologists, biochemists, and civil and environmental engineers regarding negative and positive COVID-19 impacts, respondent contributions to addressing the pandemic, and their opinions regarding COVID-19 research policies. The most common negative impact was university closures, cited by 93% of all scientists. Significant subgroup differences emerged, with higher proportions of women, assistant professors, and scientists at institutions located in COVID-19 “hotspot” counties reporting difficulties concentrating on research. Assistant professors additionally reported facing more unanticipated childcare responsibilities. Approximately half of the sample also reported one or more positive COVID-19 impacts, suggesting the importance of developing a better understanding of the complete range of impacts across all fields of science. Regarding COVID-19 relevant public policy, findings suggest divergence of opinion concerning surveillance technologies and the need to alter federal approval processes for new tests and vaccines.

## Introduction

The COVID-19 pandemic continues to dramatically impact public health and economies around the world, especially in the United States, which has disproportionately suffered from it. As of March 1, 2021, more than half a million Americans had perished due to COVID-19 (Hall, 2021). The pandemic will likely have far-reaching long-term social, economic and political consequences. The immediate effects on universities and scientific enterprises continue to be reported in the popular media and professional literature. Available evidence suggests that investigator access to on-campus university facilities and resources remains limited (Omary et al., 2020; Servick et al., 2020), the time scientists spend on research has declined sharply (Myers et al., 2020), international collaborations have been reduced (Fry et al., 2020), available resources are being diverted away from other research priorities (Kent et al., 2020; Saini et al., 2020), and peer review and other scientific standards are in danger of being compromised as scientists have rushed to confront the problem (London and Kimmelman, 2020; Schwab and Held, 2020). There is also concern that long-term COVID-19 impacts on scientific research may disproportionately fall on women (Collins et al., 2020; Cui et al., 2020; Korbel and Stegle, 2020; Minello, 2020; Squazzoni et al., 2020), persons of color (Gould and Wilson, 2020; Staniscuaski et al., 2021; Weissman, 2020), early-career investigators (Gonzales and Griffin, 2020; Kent et al., 2020; Termini and Traver, 2020; Yan, 2020), those with childcare responsibilities (Krukowski et al., 2021; Langin, 2020; Myers et al., 2020; Watchorn and Heckendorf, 2020), and graduate students (Chirikov et al., 2020; Johnson et al., 2020; Toronto Science Policy Network, 2020). Much of this information is anecdotal or comes from surveys conducted using non-probability sampling methods or unclear sample frames.

The pandemic has also generated new monitoring, diagnostic, and vaccine research aimed at controlling the spread and minimizing the severity of the disease. While advances in innovations and vaccine trials raise hopes, they have also exposed concerns that surveillance technologies could jeopardize privacy rights (Halpern, 2020; Schwartz, 2020) and expedited governmental review of new vaccines could jeopardize public health (Thorp, 2020). Although these topics have been discussed widely in the media, we know little about how the scientific community views these policy trade-offs.

We report findings from a probability-based sample survey of 362 U.S. university-based biologists, biochemists, and civil and environmental engineers concerning the impacts of the COVID-19 pandemic on their scientific productivity and investigate differences in impacts by gender, rank, and COVID-19 “hotspot” status.

## Methods

### Survey design

A national survey of scientists and engineers on the impacts of COVID-19 on academic research was conducted by the Center for Science, Technology and Environmental Policy Studies at Arizona State University. The survey instrument was developed by the author team in March 2020. A total of 67 questions were included in the final questionnaire, which respondents completed in an average of 21.1 min (SD = 2.4). The questionnaire included sections asking about impacts on scientist research (39 items), participation in COVID-19 research (5 items), opinions regarding COVID-19 research (10 items) and impacts (8 items), and personal exposure to COVID-19 (5 items). The final questionnaire items, approved by Institutional Review Boards at Arizona State University and at the University of Illinois at Chicago, are listed in the Supplemental materials.

### Sample design

A one-stage cluster sample was designed with the following protocol. We identified all R1 Carnegie classified research-intensive institutions (131 total) using the most recent Carnegie listings. We then stratified the institutions by eight Carnegie region classifications. Because the eight regions vary in size, we randomly selected 20 universities representing a proportionate distribution from each region, ensuring that selected universities within each represented multiple states. For each selected university, we developed a list of all non-tenure track, tenured, and tenure-track faculty in biology, civil and environmental engineering, and biochemistry and genomics departments. These faculty served as the sample for the survey.

### Survey administration

The online survey was administered in May 2020, in English using Sawtooth Software^®^. A total of 1968 individuals were invited to participate in the survey via email invitations with a series of personalized follow-up email reminders. Electronic prenotification messages were sent in late April prior to the survey launch. A survey invitation with a unique ID, passwords, and hyperlinks to the questionnaire was sent on May 7, followed by three reminder messages. The final completion was obtained on May 28, resulting in 362 complete responses, with an AAPOR response rate (RR4) of 20.9% (American Association for Public Opinion Research, 2016). The completed sample was weighted by gender and academic field to represent the population as closely as possible. A conservative measure of sampling error for questions answered by the full sample is ±5 percentage points.

### Geographic hotspots

Using data from the Centers for Disease Control and Prevention (CDC) (Oster et al., 2020), we identified and coded universities in the sample as to whether or not the county in which they were located was classified as a COVID-19 “hotspot” between March 8 and May 31, 2020—the dates our survey was fielded. CDC defined a “hotspot” as a county that meets criteria relating to relative temporal increases in the numbers of cases during the time period examined. More than half of all respondents (56.6%) were employed at the 10 universities in our sample that were identified as being located in hotspot counties.

### Method

Descriptive statistics are employed to present findings. All results are weighted. Supplementary Tables S1–S7 provide standard errors for all full sample survey estimates reported. Crosstabulations and *X*^2^ tests are used to compare survey responses by gender, academic rank and hotspot status. To adjust for multiple comparisons, we only report test findings for *p* < 0.001.

## Negative impacts of COVID-19 on scientists

**Fig. 1:**
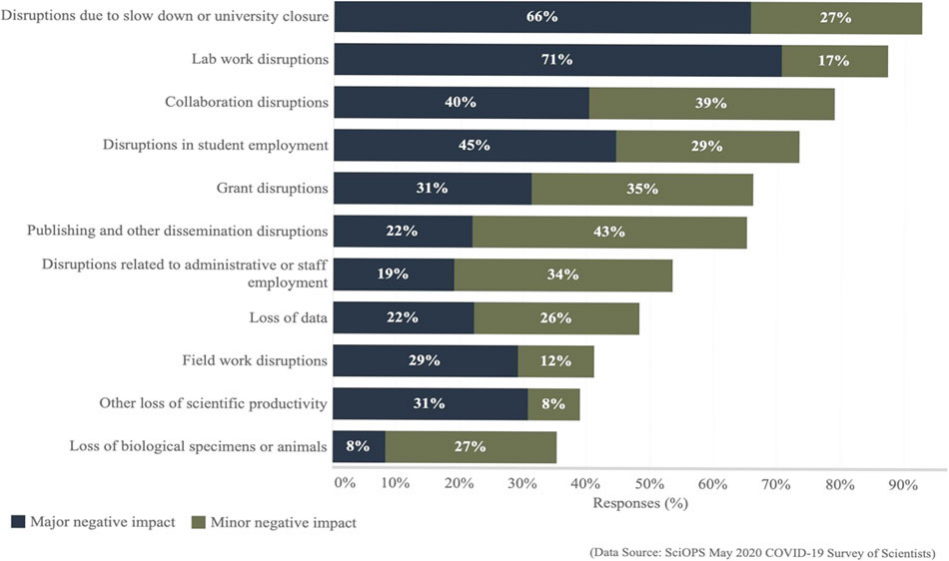
Major and minor negative impacts on academic science from COVID-19 and related policies. This chart summarizes the weighted proportions of scientists (on the *x*-axis; ranging from 0% and 100%) who identified various major (dark blue) and minor (green) impacts of social distancing and other COVID-19-related policies (listed on the *y*-axis) on their research activities. (Exact question wording: “Have social distancing and other COVID-19-related policies had a negative impact on your research in any of the following ways?”).

In response to COVID-19 in March 2020, universities were some of the first organizations to shut down in the U.S.—sending faculty, employees, and students home to work remotely. We asked our sample about the major and minor negative and positive impacts of COVID-19 work from home orders. Figure 1 shows the proportion of respondents indicating negative impacts (additional details provided in Supplementary Table S1). Looking first at major negative impacts, the most common was lab work disruptions (71%) followed by disruptions due to slow down or university closure (66%), disruptions in student employment (45%), and collaboration disruptions (40%). Figure 1 also indicates the proportions of scientists reporting minor negative impacts. The most commonly reported minor negative impacts were publishing and other dissemination disruptions (43%), collaboration (39%), grant disruptions (35%), and those related to administrative or staff employment (34%). Overall, the most common impact, major or minor, was slow down or university closure, reported by 93% of the sample.

More than one-quarter of all scientists (29%) reported they had one or more research grants facing financial problems that were directly caused by the COVID-19 pandemic. Of those reporting financial problems, two-thirds (67%) were delaying the start of data collection, 50% had applied for timeline extensions, 11% had applied for supplemental funding, 35% ended data collection early, 14% reported experiencing the destruction of lab specimens and/or animals, and 6% had laid-off research staff (results shown in Supplementary Table S5).

## Comparing negative impacts of COVID-19 by gender

In normal circumstances, female scientists face more family care responsibilities than men. Women report doing more household chores than male partners (O’Laughlin and Bischoff, 2005), and working mothers report more childcare responsibilities (Fox et al., 2011). These differences in workload at home, inevitably shape outcomes at work. The COVID-19 pandemic has further blurred the boundary between work and home and increased domestic caregiving responsibilities at the expense of work hours (Collins, 2020). Working from home is especially challenging for households with small children, elderly parents, and small working spaces. Many academic scientists now face increased family care responsibilities. We asked respondents if COVID-19-related stay-at-home policies were causing negative impacts related to concentration, anxiety, illness, and child and eldercare responsibilities.

**Fig. 2:**
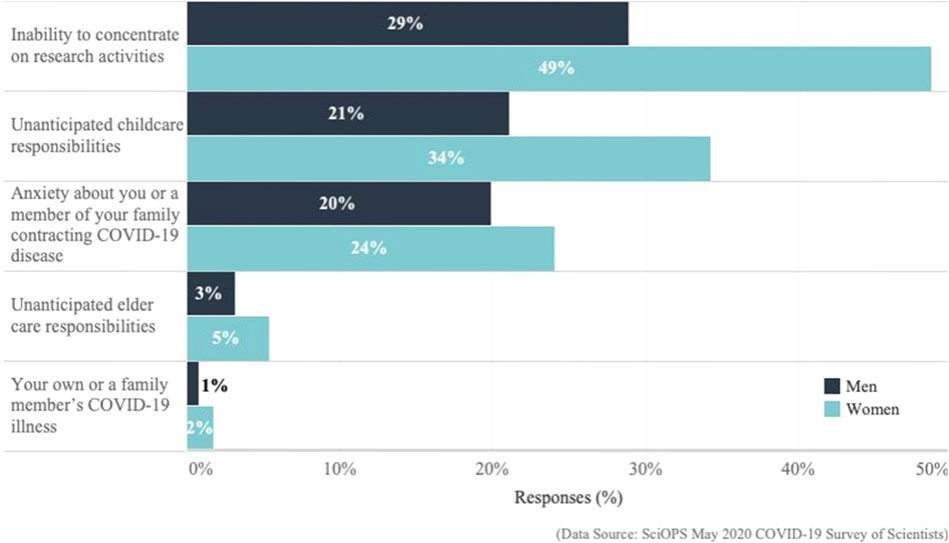
Major negative impacts of COVID-19 policies on home-life situations of scientists, by gender. This chart summarizes the weighted proportions of female (light blue) and male (dark blue) scientists (on the *x*-axis; ranging from 0% to 50%) who identified negative impacts of social distancing and other COVID-19 policies (listed on the *y*-axis) on their research activities as a consequence of their home-life situations. (Exact question wording: “Have social distancing and other COVID-19-related policies had a negative impact on your research vis-à-vis any of the following home-life situations?”).

Figure 2 and Supplementary Table S2 show major negative COVID-19 impacts on home-life situations, by gender. Men and women report the same rank order of major negative outcomes, with the inability to concentrate on research being most common. Yet, a significantly higher proportion of women report difficulties in concentrating on research (*χ*^2^ = 12.8, df = 1, *p* < 0.001). Nearly 50% of women indicated that COVID-19 stay-at-home orders extensively disturbed their research time, while less than one-third of men reported the same.

## Comparing negative impacts of COVID-19 by rank

**Fig. 3:**
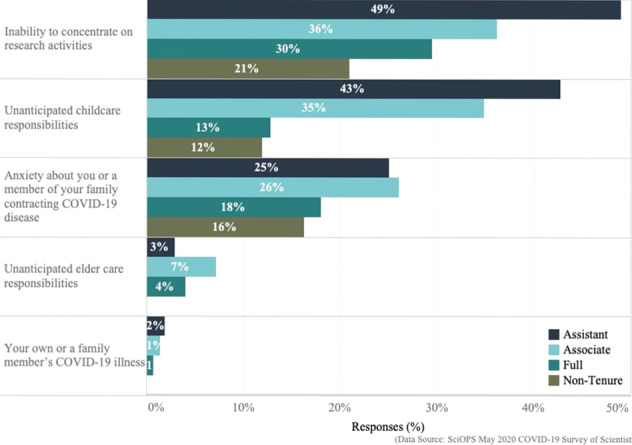
Major negative impacts of COVID-19 policies on home-life situations of scientists, by rank. This chart summarizes the weighted proportions of scientists (on the *x*-axis; ranging from 0% to 50%) by faculty rank status as assistant professor (dark blue), associate professor (light blue), full professor (green), or non-tenure track (brown) who identified negative impacts of social distancing and other COVID-19 policies (listed on the *y*-axis) on their research activities as a consequence of their home-life situations. (Exact question wording: “Have social distancing and other COVID-19-related policies had a negative impact on your research vis-à-vis any of the following home-life situations?”).

Figure 3 and Supplementary Table S2 show major negative COVID-19 impacts on home-life, by rank. Overall, faculty report similar rank order of major negative outcomes. The most common major negative impact on research was the inability to find uninterrupted time to concentrate on their research followed by unexpected childcare responsibilities and anxiety about exposure to COVID-19. Compared to all others, a significantly greater proportion of assistant professors indicated encountering childcare responsibilities (*χ*^2^ = 23.62, df = 1, *p* < 0.001) and difficulties focusing on their research (*χ*^2^ = 13.9, df = 1, *p* < 0.001) as major negative impacts.

## Comparing negative impacts of COVID-19 by hotspot status

Scientists at universities located in COVID-19 hotspot counties generally did not report experiencing disproportionate COVID-19 impacts. They were somewhat more likely to report major difficulties concentrating on research activities (22%), compared to those not situated in hotspot locations (12%), but this difference (*χ*^2^ = 4.1, df = 1, *p* < 0.05) was not statistically significant at our adjusted alpha level of *p* < 0.001.

## Positive impacts of COVID-19 on scientists

**Fig. 4:**
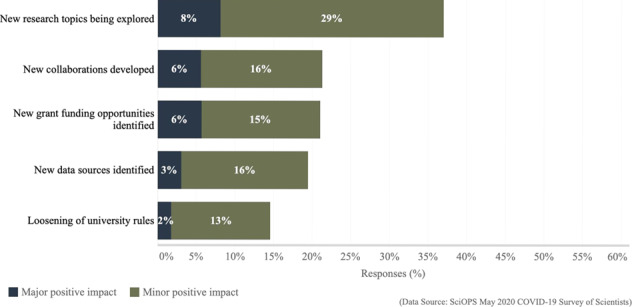
Major and minor positive impacts on academic science from COVID-19 and related policies. The chart summarizes the percent of responding scientists (on the *x*-axis; ranging from 0% to 60%) who report a major positive impact (blue) and minor positive impact (green) social distancing policies had on five aspects of respondent research. (Exact question wording: “Have social distancing policies had a positive impact on your research in any of the following ways?”).

Scientists were asked if they had experienced any positive impacts from COVID-19-related policies. Figure 4 and Supplementary Table S3 present both the major and minor positive impacts examined. In general, minor impacts were more common than major ones. The most common impact (major or minor) was the opportunity to explore new research topics, indicated by more than a third of the sample (37%). The development of new collaborations (22%), the identification of new grant funding opportunities (21%), and new data sources (19%) were each reported by approximately one in five scientists. There were no significant differences in positive impacts by gender, rank or geographic hotspot status were detected.

## Overall impacts of COVID-19 on scientists

**Fig. 5:**
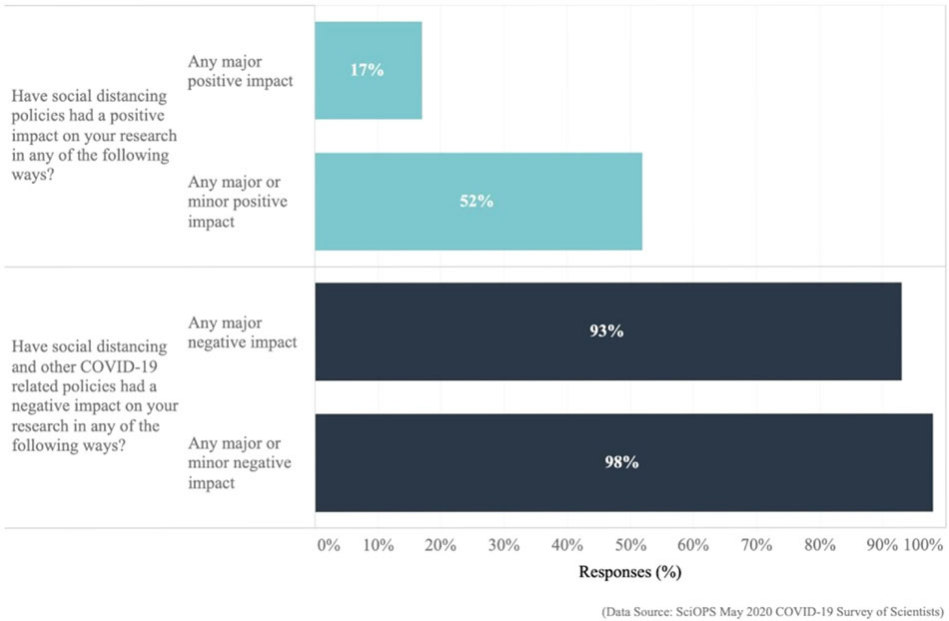
Overall reports on major and minor positive and negative impacts on academic science from COVID-19 and related policies. The chart reports the percent of responding scientists (on the *x*-axis; ranging from 0% to 100%) who report a major or major and minor positive (light blue) or major or major and minor negative impact (dark blue) from social distancing policies. (Exact question wording: (1) “Have social distancing policies had a positive impact on your research in any of the following ways?” (2) “Have social distancing and other COVID-19 related policies had a negative impact on your research vis-à-vis any of the following home-life situations?”).

Virtually all scientists (98%) reported some negative COVID-19 impacts and most reported at least one major negative impact (93%). As Fig. 5 and Supplementary Tables S1 and S3 indicate, about half also reported experiencing either a major or minor positive impact (52%), although a much smaller proportion identified a major positive impact (17%).

## Contributions to the COVID-19 pandemic response

The COVID-19 pandemic has generated opportunities for scientists to provide research expertise and resources to other researchers and to communicate with the public. We asked scientists if they had contributed expertise to help address the pandemic. Overall, 21% reported doing so. Approximately 18% indicated they had contributed to the scientific community in one or more ways, including activities such as providing lab supplies or equipment to others, collaborating on relevant experiments or analyses, or reviewing others’ research findings or reports. In addition, 13% of all scientists made COVID-19 relevant contributions to the general public by responding to media requests or helping disseminate or interpret relevant research findings. Using a conservative test for statistical difference of *p* < 0.001, no differences by gender, rank, or COVID-19 hotspot were found. These findings are reported in Supplementary Table S6.

## Perceptions about benefits and risks of COVID-19 research and technology

This public health crisis has required weighing the risks and benefits associated with the release of new technologies and scientific knowledge. Academic scientists’ opinions on research, tracking and testing policies vary widely. Just under a quarter of respondents (24%) believe the use of surveillance technology including facial recognition, fine-grained location tracking and temperature detection is necessary to mitigate the pandemic. More than half (53%) responded that surveillance technologies are ‘necessary but should be better regulated’ while another 11% reported that ‘use depends on the situation’. These results are reported in Supplementary Table S7.

**Fig. 6:**
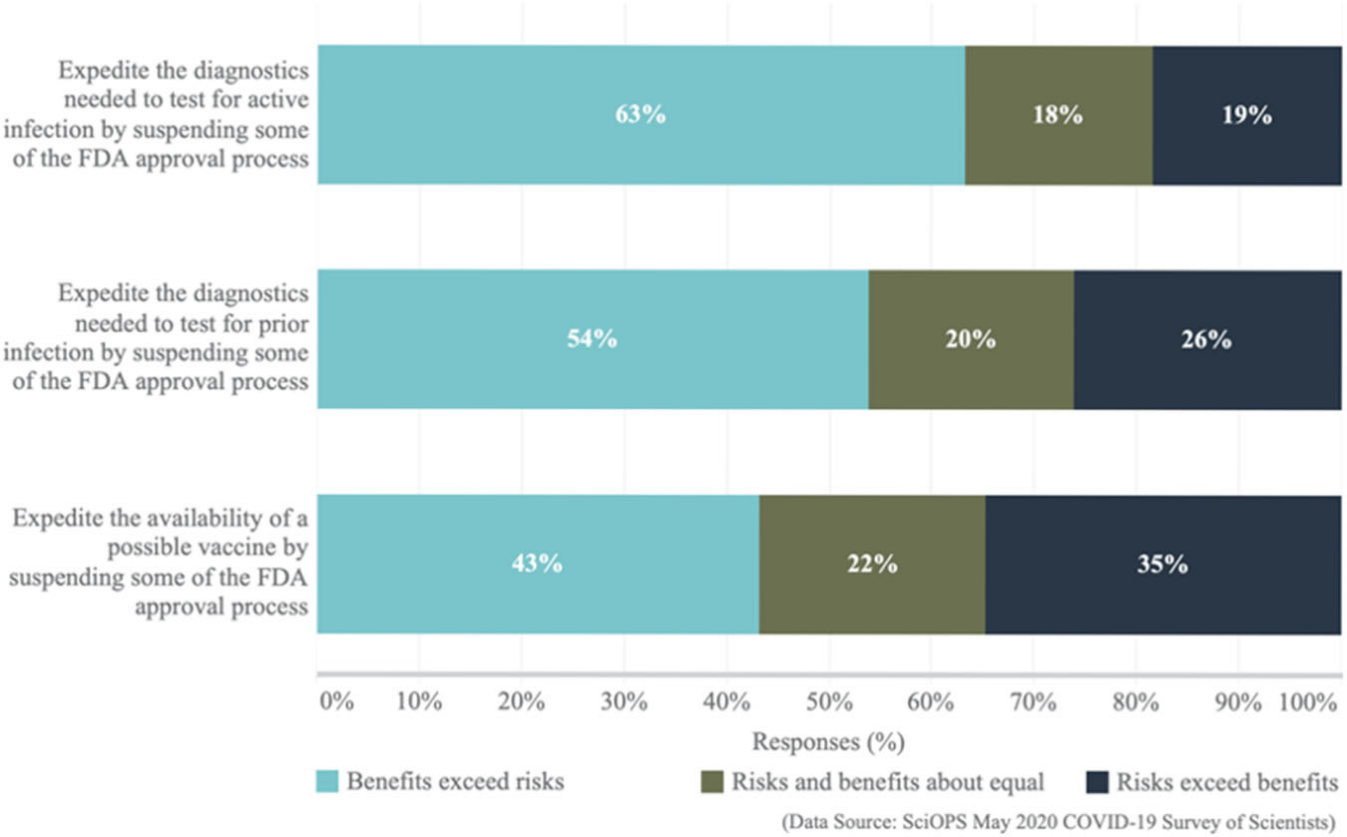
Scientist opinions regarding the approval process for COVID-19 testing and vaccine research. The chart reports the percent of responding scientists (on the *x*-axis; ranging from 0% to 100%) indicating benefits exceed risks (light blue), risks and benefits about equal (green) and risks exceed benefits (dark blue) in response to three questionnaire items about FDA approval processes for diagnostic tests and vaccine approval. (Exact question wording: “When confronted with a pandemic such as the COVID-19 disease, decisions must be made as to whether or continue following established policies for obtaining FDA approval for newly developed tests and vaccines, or to forego some established procedures in hopes of more quickly releasing products that help confront the crisis. This requires a careful balancing of the risks and benefits of these alternatives. Using the COVID-19 disease as an example, what do you believe is the risk/benefit tradeoff associated with each of the following potential decisions that might need to be made during the time of a national pandemic emergency?”).

Figure 6 and Supplementary Table S4 show that a majority of scientists believe that the benefits of expedited availability of new testing technologies during the pandemic exceed the risks. Respondents indicated that the benefits of suspending some of the Federal Drug Administration (FDA) approval processes outweigh the risks for expediting active infection testing diagnostics (63%) and prior infection testing diagnostics (54%). Yet, opinions about suspending some of the FDA approval processes to expedite the availability of a vaccine were more balanced with 43% believing the benefits outweigh the risks and 35% believe the risks outweigh the benefits. This split of opinion is reinforced by responses to a question on the ethics of bypassing some formal approval processes to distribute a vaccine more quickly (see Supplementary Table S8). Nearly the same percentage of respondents believe bypassing is unethical (31%) and ethical (29%), while 40% indicate that it would depend on the situation.

## Investigation of potential nonresponse bias

Given the survey’s response rate of 20.9%, we conducted a nonresponse bias analysis to determine if any population subgroups might be systematically over-represented or under-represented in these data. Table 1 presents findings from this analysis. Columns 1 and 2 compare survey respondents (*n* = 362) to our full sample (*n* = 1968), suggesting that females and assistant professors were over-represented in the final sample by 5.7 and 5.9 percentage points, respectively. Associate and full professors were under-represented by 3.1 and 2.6 percentage points, respectively, and participation among non-tenure-track faculty and scientists working at institutions located in hotspot areas were approximately equal to their representation in the population (i.e., 0.5 percentage point difference or less). Weighting the survey data (column 3) improved the representation of all subgroups, with the exception of scientists working at hotspot institutions, which became slightly more under-represented. Overall, the weighted sample provides a fairly close representation of the population from which it was selected.

**Table 1:** Results of nonresponse bias analysis.

Variables	Survey respondents (*n* = 362) (%)	Full sample (*n* = 1968) (%)	Weighted survey respondents (*n* = 362) (%)
Female	36.5	30.8	30.8
Assistant Professor	30.1	24.2	28.3
Associate Professor	18.8	21.9	19.0
Full Professor	39.8	42.4	41.4
Non-tenure track	11.3	11.5	11.3
In hotspot counties	54.4	53.9	56.3

## Discussion

Using a representative sample of academic scientists in U.S. research institutions, we provide evidence of both positive and negative COVID-19 impacts on science, with the negative impacts outpacing the positive. More than 90% of all scientists have experienced at least one major negative impact from COVID-19 and related policies on their research. There are stark differences in negative impacts of COVID-19 by gender and rank. Women are significantly more likely than men to report an inability to concentrate on research activities as a result of COVID-19 stay-at-home orders. Previous research indicates academic women in all fields—particularly those with children at home—will likely experience research slowdowns and produce fewer grant submissions and publications during and after the pandemic (Cui et al., 2020; Squazzoni et al., 2020; Viglione, 2020). These differentials may persist beyond the pandemic and throughout their careers.

We also find that assistant professors report significantly higher negative impacts than tenured faculty related to childcare responsibilities. This finding makes sense given assistant professors are typically younger than tenured faculty and in their prime reproductive years, thus more likely to have young children at home (Cardel et al., 2020). Additionally, junior women often outnumber senior women in STEM fields, potentially resulting in important negative cohort effects. COVID-19 has exacerbated domestic burdens and childcare responsibilities for early-career female scientists and will have inevitably negatively impact future research productivity, research funding, and tenure and promotion (Cardel et al., 2020). It will be increasingly important for universities and science funders to address these gender and rank differences in COVID-19 experiences and their differential impacts on stress, anxiety, and academic outcomes (e.g., teaching, research, and grant getting) (Gonzales and Griffin, 2020; Weissman, 2020). Cardel et al. (2020) suggest extending research periods, developing “women-only” and early career funding opportunities, providing childcare resources and support, and monitor service and teaching loads for early career researchers and women among other options. These formal efforts will be necessary to ensure gender equity gains in the academic workforce over the previous decades are not lost because of COVID-19.

Despite the publication of more than 10,000 new papers related to COVID-19 per month (Chen et al., 2021), at the time of this study previous research on the positive impacts of COVID-19 and related policies on research activity had not been reported. Indeed, half of the scientists participating in this probability survey reported one or more positive impacts. This is testimony to the resilience and innovativeness of the scientific community and suggests the need for a better understanding of the complete range of short- and long-term negative and positive impacts across all fields of science.

Regarding technology and regulatory policy, our findings reveal important divergence of opinion within the scientific community. Academic scientists do not speak with one mind on the benefits and risks of suspending federal regulatory policies for new COVID-19 technologies and vaccines. Scientists are decidedly split on the ethics of bypassing formal approval processes for expediting a COVID-19 vaccine. This is evidence of both the diversity of opinion within the academic scientific workforce and the complexity of the policy area in which trade-offs are assessed from multiple perspectives.

Although based on probability sampling, we acknowledge this report is limited to biologists, biochemists, and civil & environmental engineers only. Moreover, while we present findings by gender, because the survey did not ask about race and ethnicity, we are unable to assess differences by race or ethnicity. We know that COVID-19 has differently affected communities of color and the negative employment and wage impacts have disproportionately affected women of color both inside and outside of academia (Bohn et al., 2021; Gould and Wilson, 2020; Staniscuaski et al., 2021; Weissman, 2020). It is also important to recognize that COVID-19 is an equal opportunity pandemic, impacting research across all academic disciplines, well beyond just the STEM fields, and it is likely to affect career advancement across the entire academic community for years to come. Future research into the effects of COVID-19 policies on the scientific and academic workforce should be expanded to cover other fields of inquiry, race and ethnicity, and the intersection of race and gender.

## Supplementary information

Supplementary materials

## Data Availability

The datasets generated and analyzed during the current study are available in the Harvard Dataverse repository, 10.7910/DVN/PINEER.
