# Correction: COVID-19 and the academy: opinions and experiences of university-based scientists in the U.S.

**DOI:** 10.1057/s41599-021-00963-y

**Published:** 2021-11-15

**Authors:** Timothy P. Johnson, Mary K. Feeney, Heyjie Jung, Ashlee Frandell, Mattia Caldarulo, Lesley Michalegko, Shaika Islam, Eric W. Welch

**Affiliations:** 1 Department of Public Administration, University of Illinois at Chicago, Chicago, IL USA; 2 Center for Science, Technology and Environmental Policy Studies, School of Public Affairs, Arizona State University, Phoenix, AZ USA

**Keywords:** Sociology, Science, technology and society

Correction to: *Humanities and Social Sciences Communications* 10.1057/s41599-021-00823-9, published online 17 June 2021.

The original paper lacked a clear statement on ethical approval and informed consent. The following statements have now been added to the paper to provide additional clarity for the reader:

**Ethical approval**

The questionnaire and methodology for this study was approved by the Human Research Ethics committees at Arizona State University (Study #00011868) and at the University of Illinois at Chicago (Protocol #2020-0470).

**Informed consent**

All individuals invited to participate in the study were provided a statement of informed consent. The informed consent language clearly explained their rights as a research subject/participant. By entering the survey individuals affirmed their consent.

