# Supplementary material for: COVID-19 and the academy: opinions and experiences of university-based scientists in the U.S
Source: Humanit Soc Sci Commun. 2021 Jun 17;8(1):146. doi: 10.1057/s41599-021-00823-9 (PMC8593394; doi:10.1057/s41599-021-00823-9)
Supplement: Supplementary file 1 — Supplementary materials [file 41599_2021_823_MOESM1_ESM.pdf]

Supplementary Materials for

COVID-19 and the Academy:
Opinions and Experiences of University-based Scientists in the U.S.

This PDF file includes:

Supplementary Tables S1 to S8
Survey Instrument

Variables	(n)	Major negative impact	Minor negative impact	No negative impact	Not applicable
Loss of data	356	22.1% (2.2)	26.3% (2.4)	43.5% (2.7)	8.1% (1.5)
Loss of biological specimens or animals	355	8.4% (1.4)	26.9% (2.4)	38.5% (2.7)	26.2% (2.5)
Field work disruptions	357	29.2% (2.5)	12.0% (1.8)	15.6% (2)	43.2% (2.7)
Lab work disruptions	360	70.6% (2.5)	16.6% (2.1)	5.3% (1.3)	7.4% (1.4)
Collaboration disruptions	358	40.2% (2.7)	38.7% (2.7)	16.9% (2)	4.2% (1)
Grant disruptions	360	31.2% (2.5)	35.0% (2.6)	25.0% (2.4)	8.8% (1.5)
Publishing and other disseminations	359	22.0% (2.2)	43.2% (2.7)	31.8% (2.6)	3.0% (0.9)
Disruptions in student employment	359	44.5% (2.7)	28.8% (2.5)	19.0% (2.1)	7.7% (1.4)
Disruptions related to administrative or staff employment	358	19.1% (2.1)	34.5% (2.6)	29.8% (2.5)	16.7% (2)
Disruptions due to slow down or university closure	361	65.7% (2.6)	27.0% (2.4)	5.1% (1.2)	2.3% (0.7)
Other loss of scientific productivity	225	30.8% (3.1)	8.2% (1.8)	8.1% (1.9)	53.0% (3.4)
Any major negative impact	362	92.8% (1.3)			--
Any major or minor negative impact	362	98.1% (0.7)			--

Table S1. Major and minor negative impacts on academic science from COVID-19 and related policies: Weighted % (SE). (Exact question wording: "Have social distancing and other COVID-19 related policies had a negative impact on your research in any of the following ways?").

Variables	(n)	Females	Males	Assistant	Associate	Full	Non-Tenured
Unanticipated childcare responsibilities	362	34.2% (4.2)	21.1% (2.7)	42.2 (4.9)	35.2 (5.9)	12.7 (2.7)	11.4 (4.9)
Unanticipated elder care responsibilities	362	5.5% (2.4)	3.4% (1.2)	3.6 (2.2)	7.5 (3.3)	3.9 (1.6)	0 (0)
Your own or a family member's COVID-19 illness	362	1.4% (1.0)	0.9% (0.7)	2.1 (1.5)	1.4 (1.4)	0.5 (0.5)	0 (0)
Anxiety about you or a member of your family contracting COVID-19 disease	362	24.4% (4.0)	20.0% (2.7)	25.4 (4.4)	26.5 (5.4)	17.6 (3.3)	16.2 (5.8)
Inability to concentrate on research activities	361	48.6% (4.5)	29.0% (3.0)	49.6 (4.9)	35.6 (5.8)	28.6 (3.8)	21.2 (6.5)
Other unanticipated complications to home-life	190	13.0% (3.9)	10.1% (2.8)	10.7 (4.2)	10.1 (4.9)	12.2 (3.7)	9.0 (6.2)

Table S2. Major negative impacts of COVID-19 policies on home-life situations of scientists, by gender: Weighted % (SE). (Exact question wording: "Have social distancing and other COVID-19 related policies had a negative impact on your research vis-à-vis any of the following home-life situations?").

Variables	(n)	Major positive impact	Minor positive impact	No positive impact	Not applicable
New collaborations developed	360	5.6% (1.4)	16.4% (2.0)	66.8% (2.6)	11.2% (1.6)
New research topics being explored	360	8.1% (1.5)	28.8% (2.5)	54.1% (2.7)	9.0% (1.5)
New grant funding opportunities identified	362	5.7% (1.3)	15.3% (2.0)	67.4% (2.6)	11.6% (1.7)
New data sources identified	360	3.1% (0.9)	16.4% (2.0)	66.2% (2.6)	14.4% (1.9)
Loosening of university rules	360	1.7% (0.7)	12.9% (1.9)	66.1% (2.6)	19.3% (2.1)
Other positive impacts	172	8.9% (2.1)	4.0% (1.4)	17.7% (3.1)	69.3% (3.6)
Any major positive impact	362	16.5% (2.1)			--
Any major or minor positive impact	362	51.8% (2.7)			--

Table S3. Major and minor positive impacts on academic science from COVID-19 and related policies: Weighted % (SE). (Exact question wording: “Have social distancing and other COVID-19 related policies had a positive impact on your research in any of the following ways?”).

Variables	(n)	Benefits exceed risks	Risks and benefits about equal	Risks exceed benefits
Expedite the diagnostics needed to test for active infection by suspending some of the FDA approval process	351	63.2% (2.7)	18.3% (2.2)	18.5% (2.1)
Expedite the diagnostics needed to test for prior infection by suspending some of the FDA approval process	350	53.8% (2.7)	20.1% (2.2)	26.1% (2.4)
Expedite the availability of a possible vaccine by suspending some of the FDA approval process	351	43.1% (2.7)	22.2% (2.3)	34.7% (2.6)

Table S4. Scientist opinions regarding approval process for COVID-19 testing and vaccine research: Weighted % (SE). (Exact question wording: "When confronted with a pandemic such as the COVID-19 disease, decisions must be made as to whether or continue following established policies for obtaining FDA approval for newly developed tests and vaccines, or to forego some established procedures in hopes of more quickly releasing products that help confront the crisis. This requires a careful balancing of the risks and benefits of these alternatives. Using the COVID-19 disease as an example, what do you believe is the risk/benefit tradeoff associated with each of the following potential decisions that might need to be made during the time of a national pandemic emergency?").

Variables	(n)	Yes	No
Do you currently have one or more research grants that are facing financial problems that are directly caused by the COVID-19 pandemic?	362	29.4% (2.5)	70.6% (2.5)
[IF YES]: have you taken any of the following actions in response to these financial problems? [SELECT ALL THAT APPLY]			
Applied for a timeline extension	108	50.2% (5.0)	49.8% (5.0)
Applied for supplemental funding	108	11.2% (3.2)	88.8% (3.2)
Laid-off research staff	108	5.7% (2.3)	94.3% (2.3)
Ended data collection early	108	35.4% (4.7)	64.6% (4.7)
Delayed the start of data collection	108	66.8% (4.7)	33.2% (4.7)
Destruction of lab specimens or animals	108	13.6% (3.3)	86.4% (3.3)
Anything else	108	22.4% (4.0)	77.6% (4.0)

Table S5. Scientist reports of grant financial problems and actions taken in response: Weighted % (SE).

Variables	(n)	Yes	No
Made contributions of expertise to scientific community	361	17.5% (2.1)	82.5% (2.1)
Made contributions of expertise to general public understanding	361	13.1% (1.8)	86.9% (1.8)
Made contributions of expertise to either scientific community or general public	361	21.1% (2.2)	78.9% (2.2)

Table S6. Scientist contributions of expertise to address COVID-19 pandemic: Weighted % (SE).

Variable	(n)	Not necessary	Necessary but should be better regulated	Necessary	Depends
How necessary do you feel the use of such technologies are for mitigating a public health crisis such as the COVID-19 pandemic?	356	12.1% (1.8)	52.8% (2.7)	24.2% (2.3)	10.9% (1.7)

Table S7. Scientist opinions regarding use of surveillance technology for disease transmission tracking: Weighted % (SE). (Exact question wording: “Some countries have been increasingly using surveillance technologies (e.g., fine grain location tracking, facial recognition, automated temperature detection etc.) to track disease transmission.”).

Variable	(n)	Would be ethical	Would not be ethical	Depends
Do you believe it would or would not be ethical to bypass some of the formal approval process in order to begin distributing any promising new COVID-19 vaccines more quickly?	354	29.0% (2.5)	30.7% (2.5)	40.4% (2.7)

Table S8. Scientist opinions regarding ethics of bypassing FDA approval process of COVID-19 vaccine development: Weighted % (SE). (Exact question wording: "There is currently no vaccine available for the COVID-19 disease. Given that the regular process for developing, testing and obtaining FDA approval for a new vaccine may require one year or longer to complete, do you believe it would or would not be ethical to bypass some of the formal approval process in order to begin distributing any promising new COVID-19 vaccines more quickly?").

Survey Instrument

==========================
Question Name: Q1aNegImpactLoss
==========================
Question: Have social distancing and other COVID-19 related policies had a <u>negative</u> impact on your research in any of the following ways?

1 Loss of data [Q1aNegImpactLoss_r1]
2 Loss of biological specimens or animals [Q1aNegImpactLoss_r2]
3 Field work disruptions [Q1aNegImpactLoss_r3]
4 Lab work disruptions [Q1aNegImpactLoss_r4]
5 Collaboration disruptions [Q1aNegImpactLoss_r5]
6 Grant disruptions [Q1aNegImpactLoss_r6]
7 Publishing and other dissemination disruptions [Q1aNegImpactLoss_r7]
8 Disruptions in student employment [Q1aNegImpactLoss_r8]
9 Disruptions related to administrative or staff employment [Q1aNegImpactLoss_r9]
10 Disruptions due to slow down or university closure [Q1aNegImpactLoss_r10]
11 Other loss of scientific productivity (please specify) [Q1aNegImpactLoss_r11]

[Scale]:
1 No negative impact
2 Minor negative impact
3 Major negative impact
4 Not applicable

==========================
Question Name: Q1bNegimpactsit2
==========================
Question: Have social distancing and other COVID-19 related policies had a <u>negative</u> impact on your research vis-à-vis any of the following <u>home-life</u> situations?

1 Unanticipated childcare responsibilities [Q1bNegimpactsit2_r1]
2 Unanticipated elder care responsibilities [Q1bNegimpactsit2_r2]
3 Your own or a family member's COVID-19 illness [Q1bNegimpactsit2_r3]
4 Anxiety about you or a member of your family contracting COVID-19 disease [Q1bNegimpactsit2_r4]
5 Inability to concentrate on research activities [Q1bNegimpactsit2_r5]
6 Other unanticipated complications to home-life (please specify) [Q1bNegimpactsit2_r6]

[Scale]:
1 No negative impact
2 Minor negative impact
3 Major negative impact
4 Not applicable

==========================
Question Name: Q2aPosImpactRsrch

===========================

Question: Have social distancing policies had a positive impact on your research in any of the following ways?

1 New collaborations developed [Q2aPosImpactRsrch_r1]
2 New research topics being explored [Q2aPosImpactRsrch_r2]
3 New grant funding opportunities identified [Q2aPosImpactRsrch_r3]
4 New data sources identified [Q2aPosImpactRsrch_r4]
5 Loosening of university rules [Q2aPosImpactRsrch_r5]
6 Other positive impacts (please specify) [Q2aPosImpactRsrch_r6]

[Scale]:
1 No positive impact
2 Minor positive impact
3 Major positive impact
4 Not Applicable

===========================

Question Name: Q3FinImpact

===========================

Question: Do you currently have one or more research grants that are facing financial problems that are directly caused by the COVID-19 pandemic? [Q3FinImpact]

1 Yes
2 No

===========================

Question Name: Q3FinImpactAct

===========================

Question: Have you taken any of the following actions in response to these financial problems? [select all that apply]

1 Applied for a timeline extension [Q3FinImpactAct_1]
2 Applied for supplemental funding [Q3FinImpactAct_2]
3 Laid-off research staff [Q3FinImpactAct_3]
4 Ended data collection early [Q3FinImpactAct_4]
5 Delayed the start of data collection [Q3FinImpactAct_5]
6 Destruction of lab specimens or animals [Q3FinImpactAct_6]
7 Anything else (please specify) [Q3FinImpactAct_7]

===========================

Question Name: Q4ExpertHelp

===========================

Question: Since January 1, 2020, have you personally contributed your expertise to help address the COVID-19 pandemic? [Q4ExpertHelp]

1 Yes
2 No

===========================
Question Name: Q4aExpertHelpHow
===========================
Question: How have you contributed your expertise to help address the COVID-19 pandemic? [select all that apply]

1 Provided lab supplies or equipment to other researchers working on COVID-19 [Q4aExpertHelpHow_1]
2 Collaborated in the conduct experiments relevant to COVID-19 [Q4aExpertHelpHow_2]
3 Collaborated to conduct analyses relevant to COVID-19 [Q4aExpertHelpHow_3]
4 Reviewed others' research findings or reports relevant to COVID-19 [Q4aExpertHelpHow_4]
5 Responded to media requests about COVID-19 [Q4aExpertHelpHow_5]
6 Helped disseminate COVID-19 relevant research findings to the public [Q4aExpertHelpHow_6]
7 Helped interpret COVID-19 relevant research findings for the public [Q4aExpertHelpHow_7]
8 Other COVID-19 relevant volunteer work (please specify) [Q4aExpertHelpHow_8]

===========================
Question Name: help_science (Note: this is a recoded variable from Q4aExpertHelpHow)
===========================
If the respondent has identified that s/he has contributed their expertise in any of the following ways: (1) Q4aExpertHelpHow_1, (2) Q4aExpertHelpHow_2, (3) Q4aExpertHelpHow_3, (4) Q4aExpertHelpHow_4

1 Yes
0 No
===========================
Question Name: help_public (Note: this is a recoded variable from Q4aExpertHelpHow)
===========================
If the respondent has identified that s/he has contributed their expertise in any of the following ways: (1) Q4aExpertHelpHow_5, (2) Q4aExpertHelpHow_6, (3) Q4aExpertHelpHow_3, (4) Q4aExpertHelpHow_7

1 Yes
0 No

===========================
Question Name: help_any (Note: this is a recoded variable from Q4aExpertHelpHow)
===========================
If the respondent has identified that s/he has contributed their expertise either for help_science or help_public

1 Yes
0 No

===========================
Question Name: Q9FDARisk
===========================

Question: When confronted with a pandemic such as the COVID-19 disease, decisions must be made as to whether to continue following established policies for obtaining FDA approval for newly developed tests and vaccines, or to forego some established procedures in hopes of more quickly releasing products that help confront the crisis. This requires a careful balancing of the risks and benefits of these alternatives.

Using the COVID-19 disease as an example, what do you believe is the risk/benefit tradeoff associated with each of the following potential decisions that might need to be made during the time of a national pandemic emergency?

1 Expedite the diagnostics needed to test for <u>active infection</u> by suspending some of the FDA approval process [Q9FDARisk_r1]
2 Expedite the diagnostics needed to test for <u>prior infection</u> by suspending some of the FDA approval process [Q9FDARisk_r2]
3 Expedite the availability of a possible vaccine by suspending some of the FDA approval process [Q9FDARisk_r3]

[Scale]:
1 Risks greatly exceed benefits
2 Risks somewhat exceed benefits
3 Risks and benefits about equal
4 Benefits somewhat exceed risks
5 Benefits greatly exceed risks

==========================
Question Name: Q11FDAbypass
==========================
Question: There is currently no vaccine available for the COVID-19 disease. Given that the regular process for developing, testing and obtaining FDA approval for a new vaccine may require one year or longer to complete, do you believe it would or would not be <u>ethical</u> to bypass some of the formal approval process in order to begin distributing any promising new COVID-19 vaccines more quickly? [Q11FDAbypass]

1 Would be ethical
2 Would not be ethical
3 Depends (please specify)

==========================
Question Name: Q12Surveillence
==========================
Question: Some countries have been increasingly using surveillance technologies (e.g., fine grain location tracking, facial recognition, automated temperature detection etc.) to track disease transmission.

How necessary do you feel the use of such technologies are for mitigating a public health crisis such as the COVID-19 pandemic? [Q12Surveillence]

1 Not necessary

2 Necessary but should be better regulated
3 Necessary
4 Depends (please specify) [Respondent Specify]

============================
Question Name: female
============================
1 Yes
2 No
